# The Autophagy-Related Protein ATG8 Orchestrates Asexual Development and AFB1 Biosynthesis in *Aspergillus flavus*

**DOI:** 10.3390/jof10050349

**Published:** 2024-05-13

**Authors:** Qingru Geng, Jixiang Hu, Pingzhi Xu, Tongzheng Sun, Han Qiu, Shan Wang, Fengqin Song, Ling Shen, Yongxin Li, Man Liu, Xue Peng, Jun Tian, Kunlong Yang

**Affiliations:** JSNU-UWEC Joint Laboratory of Jiangsu Province Colleges and Universities, School of Life Science, Jiangsu Normal University, Xuzhou 221116, China; qingru950504@163.com (Q.G.); hu030602@outlook.com (J.H.); 18281976836@163.com (P.X.); americo2333@gmail.com (T.S.); qhd163yxlove@163.com (H.Q.); ws13569294820@163.com (S.W.); songfengqin@jsnu.edu.cn (F.S.); lingshen@jsnu.edu.cn (L.S.); yongxinli@jsnu.edu.cn (Y.L.); liuman861214@163.com (M.L.)

**Keywords:** autophagy, *Aspergillus flavus*, AFB1 biosynthesis, ATG8, pathogenicity

## Abstract

Autophagy, a conserved cellular recycling process, plays a crucial role in maintaining homeostasis under stress conditions. It also regulates the development and virulence of numerous filamentous fungi. In this study, we investigated the specific function of ATG8, a reliable autophagic marker, in the opportunistic pathogen *Aspergillus flavus*. To investigate the role of *atg8* in *A. flavus*, the deletion and complemented mutants of *atg8* were generated according to the homologous recombination principle. Deletion of *atg8* showed a significant decrease in conidiation, spore germination, and sclerotia formation compared to the WT and *atg8^C^* strains. Additionally, aflatoxin production was found severely impaired in the ∆*atg8* mutant. The stress assays demonstrated that ATG8 was important for *A. flavus* response to oxidative stress. The fluorescence microscopy showed increased levels of reactive oxygen species in the ∆*atg8* mutant cells, and the transcriptional result also indicated that genes related to the antioxidant system were significantly reduced in the ∆*atg8* mutant. We further found that ATG8 participated in regulating the pathogenicity of *A. flavus* on crop seeds. These results revealed the biological role of ATG8 in *A. flavus*, which might provide a potential target for the control of *A. flavus* and AFB1 biosynthesis.

## 1. Introduction

*Aspergillus flavus*, a saprophytic soil fungus, infects insects and contaminates both preharvest and postharvest seed crops such as maize and peanuts, while also producing potent aflatoxins (AFs) [1,2]. Among the AFs, aflatoxins B1, B2, G1, and G2 are the major four and aflatoxin B1 (AFB1), the most toxic and potent hepatocarcinogenic compound among them, primarily targets the liver [3,4]. Chronic low-level exposure to AFB1 can lead to immunosuppression and hepatocellular carcinoma, while a single acute exposure could be fatal [5,6]. Additionally, *A. flavus*, the second most common cause of aspergillosis after *Aspergillus fumigatus*, poses a significant threat to immunocompromised individuals [7,8,9]. Sino-orbital, cerebral, and ophthalmic infections due to *A. flavus* are the major clinical types in aspergillosis, after pulmonary aspergillosis [10]. It has emerged as a predominant pathogen in fungal sinusitis and keratitis globally [11]. Aflatoxins and *Aspergillus* not only devalue contaminated crops and cause significant agricultural economic losses but also pose a substantial threat to human health [12,13]. Therefore, tackling the problem of *A. flavus* and aflatoxin contamination is a pressing and crucial endeavor.

Numerous management strategies have been developed to address *A. flavus* contamination. One commonly employed approach is improving storage conditions [14]. Moreover, the use of anti-mildew agents is crucial for preventing fungal contamination. For instance, quercetin has been found to effectively inhibit the proliferation of *A. flavus* in a dose-dependent manner [15]. Research has shown that the growth of *A. flavus* hyphae and the germination of its spores can be suppressed in the presence of α-Fe_2_O_3_ nanorods under sunlight irradiation [16]. Furthermore, p-anisaldehyde (AS) has been recognized as a natural antifungal agent against *A. flavus*, with a demonstrated ability to modulate AFB1 biosynthesis [17]. In addition, biological control methods, including the use of *Trichoderma* strains, offer the potential for reducing *A. flavus* and its toxin levels through enzymatic degradation or complexation [18]. Despite substantial efforts, *A. flavus* and AF prevention and control remain significant global challenges. Hence, investigating the growth and development processes of *A. flavus* in its host, along with the regulatory mechanisms of aflatoxin synthesis, will offer critical insights and a theoretical framework for effectively managing *A. flavus* contamination.

Autophagy is a cellular degradation process in which cytosolic components and organelles are enclosed within double-membrane vesicles known as autophagosomes. These autophagosomes subsequently merge with degradative organelles, such as the vacuole/lysosome, where the enclosed contents are broken down and the resulting macromolecules are recycled [19,20]. ATG8, a ubiquitin-like protein, binds with the lipid phosphatidylethanolamine (PE) to form Atg8-PE, a crucial component for the formation of double-membrane autophagosomes [21]. Consequently, ATG8 is considered a reliable autophagic marker for monitoring autophagy progression in cells at both microscopic and molecular levels [22]. The molecular mechanisms of autophagy have been extensively investigated in yeast, where autophagy initiation, cargo recognition, cargo engulfment, and vesicle closure depend on ATG8 [23]. Furthermore, autophagy plays crucial roles in various aspects of filamentous fungi biology, including asexual and sexual development, nutrient deprivation responses, cellular stress responses, and pathogenicity. In *Magnaporthe oryzae*, mutant strains with impaired autophagy exhibit severely reduced or completely lost pathogenicity [24,25], along with decreased production of asexual spores [26]. Autophagy also plays a critical role in the growth, development, and pathogenicity of *Fusarium graminearum* [27] and *Fusarium oxysporum* [28]. Significantly, its potential role in mycotoxin biosynthesis has been reported in *F. graminearum* [29,30].

While the function of ATG8 has been reported in other fungi, its specific role in *A. flavus* remains uncharacterized. In this study, we identified ATG8 in *A. flavus* and examined its involvement in hyphal growth, sporulation, sclerotia development, stress tolerance, aflatoxin production, and pathogenicity. These findings will provide valuable insights for the development of potential novel control strategies.

## 2. Materials and Methods

### 2.1. Strains and Culture Conditions

The *A. flavus* strains utilized in this research are described in Table 1. All strains were inoculated onto potato dextrose agar (PDA) medium and incubated at 37 °C for 5 days. Spores were gathered employing a 0.001% Tween-20 solution, quantified with a hemocytometer, and the spore concentration was adjusted to 10^6^ spores/mL for subsequent analysis.

### 2.2. Bioinformatic Identification and Analysis of ATG8

ATG8 sequences from various species were obtained by querying the GenBank reference proteins database (https://www.ncbi.nlm.nih.gov/, accessed on 25 December 2021), which included organisms such as *A*. *flavus*, *Ceratocystis fimbriata*, *Saccharomyces cerevisiae*, *A*. *oryzae*, *Homo sapiens*, *Mus musculus*, *A*. *fumigatus*, *Pyricularia oryzae*, *A*. *alternata*, *F*. *graminearum*, *Neurospora crassa*, and *Drosophila melanogaster*. A phylogenetic tree was constructed for these proteins using MEGA 7.0. The domains in ATG8 were visualized using DOG 2.0. Furthermore, the three-dimensional structure of the ATG8 protein was modeled utilizing the SWISS-MODEL homology modeling server (https://swissmodel.expasy.org/, accessed on 6 March 2022).

### 2.3. Strain Construction

The deleted and complemented mutants of *atg8* were constructed according to the homologous recombination principle. To generate the *atg8* deleted strain (Δ*atg8*), flanking regions (5′ and 3′) of the *atg8* gene, along with *argB* sequences, were amplified from wild-type genomic DNA using the primers detailed in Table 2. Subsequently, these three fragments were fused by PCR, and the resulting PCR products were then introduced into the protoplasts of the parental strains TJES20.1 and TXZ21.3 following the previously described approach [32]. The upstream homologous arm fragment (AP), downstream homologous arm fragment (BP), and open reading frame (ORF) were amplified from the genomic DNA of selected transformants using three primer pairs (*atg8*/p1 and *argB*/170R, *argB*/1205F and *atg8*/p6, *atg8*-orf/F and *atg8*-orf/R), respectively. This procedure yielded Δ*atg8* and Δ*atg8*Δ*pyrG* mutants.

To generate the *atg8* complemented strain (*atg8*^C^), the upstream region (comprising 5′ flanking regions and the coding sequence of *agt8*) and the downstream region (containing *argB* sequences) were amplified from wild-type genomic DNA. Subsequently, the purified PCR products were fused with the *A. fumigatus pyrG* gene. The resulting fused fragment was then introduced into protoplasts of the Δ*atg8*Δ*pyrG* mutant to generate the *atg8* complemented strain. Confirmation of all transformants was performed via PCR. The transformants were tested by amplifying AP, BP, and ORF fragments with three pairs of primers (c-*atg8*/p1 and *pyrG*/801R, *pyrG*/351F and c-*atg8*/p6, *atg8*-orf/F and *atg8*-orf/R), respectively. Those *atg8*^C^ transformants containing the AP, BP, and ORF fragments were successfully generated.

### 2.4. Phenotype Analysis

To analyze the growth phenotypes of mutant strains on various media, the WT, Δ*atg8*, and *atg8*^C^ strains were incubated in the dark at 37 °C on potato dextrose agar (PDA, HuanKai Microbial, Guangzhou, China), glucose minimal media (GMM, 10 g/L glucose, 6 g/L NaNO_3_, 1.52 g/L KH_2_PO_4_, 0.52 g/L MgSO_4_·7H_2_O, 0.52 g/L KCl, and 1 mL/L trace elements), yeast extract glucose tryptone agar (YGT, 5 g/L yeast extract, 20 g/L glucose, and 1 mL/L trace elements), and yeast extract sucrose agar (YES, 20 g/L yeast extract, 150 g/L sucrose, and 1 g/L MgSO_4_·7H_2_O) solid culture media for 5 days. Following incubation, colony diameters were measured. Spores were collected using 0.001% Tween 20 and counted by a hemocytometer. To evaluate conidial germination rates, 1 μL of spore solution (10^6^ spores/mL) was spot inoculated onto 1% agar medium and incubated at 37 °C for 3, 6, 9, and 12 h. Spore germination was then observed using an inverted optical microscope. For sclerotium analysis, the spore suspension was inoculated onto glucose minimal sorbitol media (GMMS, 10 g/L glucose, 6 g/L NaNO_3_, 1.52 g/L KH_2_PO_4_, 0.52 g/L MgSO_4_·7H_2_O, 0.52 g/L KCl, 1 mL/L trace elements, and 20 g/L sorbitol) solid medium, cultivated at 37 °C for 5 days, and the sclerotia were subsequently counted.

### 2.5. Aflatoxin B1 Extraction and Determination

For aflatoxin detection, 10^6^ spores were inoculated into 8 mL of the YES liquid culture medium. After 5 days of incubation at 29 °C, 800 μL of the culture was mixed with an equal volume of dichloromethane, followed by centrifugation. Then, 700 μL of the lower liquid phase was collected and air-dried. Following drying, the tube walls were rinsed with 20 μL of dichloromethane, and the remaining 10 μL of the sample was analyzed using thin-layer chromatography (TLC) on a silica gel plate. AFB1 standard solution (Shanghai yuanye Bio-Technology Co., Ltd., Shanghai, China) was used as the control. The TLC results were recorded using a UV gel imaging system (BIO RAD ChemiDoc XRS, Hercules, CA, USA). Finally, Image J 1.8.0 was utilized to quantify the spots.

### 2.6. Stress Analysis

To investigate the role of ATG8 in *A. flavus* response to environmental stress, 2 μL of spore suspension was inoculated onto GMM solid medium supplemented with various stress-inducing agents: oxidative stress agents (6 mM and 8 mM H_2_O_2_), cell membrane stress agents (0.005% and 0.01% Sodium dodecyl sulfate, SDS), cell wall stress agents (200 μg/mL and 300 μg/mL Calcofluor white (CFW); 200 μg/mL and 300 μg/mL Congo red, CR), and high osmotic stress media (1 M and 2 M KCl, 1 M and 2 M NaCl). The cultures were then incubated at 37 °C in darkness for 5 days. Afterward, colony diameters were measured, and inhibition rates were calculated.

### 2.7. ROS Measurement

To elucidate the impact of ATG8 on the levels of reactive oxygen species in *A. flavus*, 100 μL spore suspension (10^6^ spores/mL) was inoculated into 900 μL of PDB medium and incubated at 37 °C for 12 h. Following this, *A. flavus* mycelia were harvested by centrifugation and washed 2–3 times with PBS. The mycelia were then treated with 10 μM 2′-7′-dichlorofluorescein diacetate (DCFH-DA) at 37 °C for 30 min in darkness and washed with PBS. Subsequently, fluorescence microscopy (Leica, Cellvizio system DualBand, Heidelberg, Germany) was employed to analyze the samples (Excitation = 488 nm; emission = 525 nm).

### 2.8. Seed Infections

In order to investigate the impact of ATG8 on the pathogenicity of *A. flavus*, peanut, and maize kernels were utilized in infection experiments. The peanut and maize kernels were first sterilized using 1% sodium hypochlorite and then rinsed three times with sterile water. Subsequently, they were sterilized with 75% ethanol and rinsed again three times with sterile water. Following this, five seeds were placed in a sterile culture dish, and each seed was inoculated with 10 μL of spore suspension (10^6^ spores/mL). After a 5-day incubation period, infected seeds were assessed for conidia and aflatoxin production.

### 2.9. Real-Time Quantitative Reverse Transcription PCR

To evaluate the gene expression of the *brlA* pathway, *A. flavus* strains were grown in PDB at 37 °C under static liquid conditions for 48 h. To assess gene expression associated with aflatoxin production, strains were cultured in YES medium at 29 °C with shaking at 200 rpm for 48 h. For other RT-qPCR analyses, strains were cultured in PDB at 37 °C with shaking at 200 rpm for 48 h. The mycelium was collected and ground with liquid nitrogen. Subsequently, TRIzol (Invitrogen, Carlsbad, CA, USA) was employed to extract total RNA following the manufacturer’s instructions. After RNA isolation, nanophotometer UV/Vis spectrophotometer (Implen, NanoPhotometer^®^ N50, Stuttgart, Germany) was employed to check the quality and quantity of RNA. cDNA was synthesized using a RevertAid First Strand cDNA Synthesis Kit (Thermo Fisher Scientific, Waltham, MA, USA). The TransStart Top Green qPCR SuperMix (TransGen Biotech, Beijing, China) was used in the Real-time PCR System (Thermo Fisher Scientific, Waltham, MA, USA). Primers for gene expression detection are listed in Table 3, with the actin gene used as a reference in this experiment.

### 2.10. Statistical Analysis

In this study, all experiments were conducted independently at least three times. Error bars represent standard deviation. Statistical analyses employed two-tailed unpaired Student’s *t*-test, two-way analysis of variance, followed by a multiple comparison test with Geisser–Greenhouse correction in GraphPad Prism 9, unless otherwise specified. Statistical significance was indicated by asterisks: * *p* < 0.05, ** *p* < 0.01, *** *p* < 0.001.

## 3. Results

### 3.1. Identification of ATG8 Protein in A. flavus and Prediction of the Tertiary Structure of ATG8 Protein

**Figure 1:**
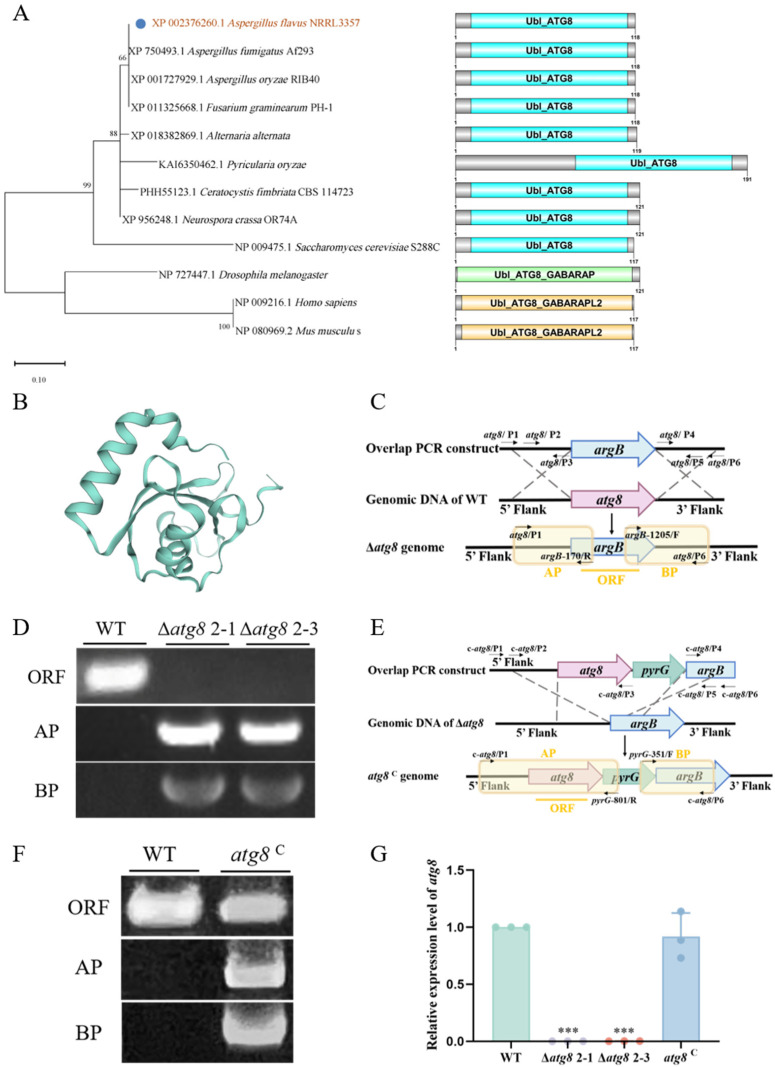
The bioinformatics analysis of ATG8 protein and the construction of *A. flavus* mutants. (**A**) Phylogenetic tree analysis of ATG8 orthologs from twelve species (*A. flavus* NRRL3357, *A. fumigatus* Af293, *A. oryzae* RIB40, *F. graminearum* PH-1, *A. alternata*, *P. oryzae*, *C. fimbriata* CBS 114723, *N. crassa* OR74A, *S. cerevisiae* S288C, *Drosophila melanogaster*, *Homo sapiens*, *Mus musculus*) and domain identification of ATG8 visualized by DOG 2.0; (**B**) Prediction of the tertiary structure of *A. flavus* ATG8 using SWISS-MODEL (https://swissmodel.expasy.org/, accessed on 6 March 2022); (**C**) Construction diagram of the Δ*atg8*; (**D**) PCR verification using genomic DNA from WT and Δ*atg8* strains, ORF: open reading frame of the *atg8* gene, AP: upstream homologous arm fragment, and BP: downstream homologous arm fragment; (**E**) Construction diagram of the *atg8*^C^; (**F**) PCR verification using genomic DNA from WT and *atg8*^C^ strains; (**G**) Relative expression levels of *atg8* in WT, Δ*atg8*, and *atg8*^C^ strains. *** indicates *p* < 0.001.

To elucidate the biofunction of ATG8 in *A. flavus*, we acquired the amino acid sequence of XP_002376260.1 from NCBI (http://www.ncbi.nlm.nih.gov/, accessed on 25 December 2021). Additionally, the amino acid sequences of ATG8 from various species (*C. fimbriata*, *S. cerevisiae*, *A. oryzae*, *Homo sapiens*, *Mus musculus*, *A. fumigatus*, *P. oryzae*, *A. alternata*, *F. graminearum*, *N. crassa*, *Drosophila melanogaster*) were retrieved through BLAST. Subsequently, a phylogenetic analysis of ATG8 proteins was conducted using MEGA 7.0. The analysis revealed that *A. flavus* ATG8 exhibited significant similarity to orthologs of *A. fumigatus* Af293, *A. oryzae* RIB40, and *F. graminearum* PH-1. Domain analysis revealed that the XP_002376260.1 protein contains the conserved domain Ubl_ATG8, showing structural similarity to known and validated ATG8 proteins (Figure 1A). Therefore, we named the XP_002376260.1 protein ATG8. Furthermore, the predicted tertiary structure of *A. flavus* ATG8 was successfully generated using SWISSMODEL (https://swissmodel.expasy.org/, accessed on 6 March 2022) (Figure 1B).

### 3.2. Deletion and Complementation of atg8 in A. flavus

The *atg8* deletion and complementation mutants were created through homologous recombination (Figure 1C,E). The diagnostic PCR and RT-qPCR analysis were employed to confirm the successful construction of mutant strains. As illustrated in Figure 1D, AP and BP were obtained from the genomic DNA of the Δ*atg8* strain, while the ORF could not be amplified. Conversely, AP, BP, and ORF fragments were successfully amplified from the genome of the *atg8*^C^ strain (Figure 1F). Subsequent RT-qPCR analysis further validated the deletion of the atg8 gene in Δ*atg8* and its restored expression level in *atg8*^C^ (Figure 1G). These findings indicate the successful construction of the Δ*atg8* and *atg8*^C^ strains.

### 3.3. ATG8 Is Crucial for Hyphal Growth and Conidiation in A. flavus

**Figure 2:**
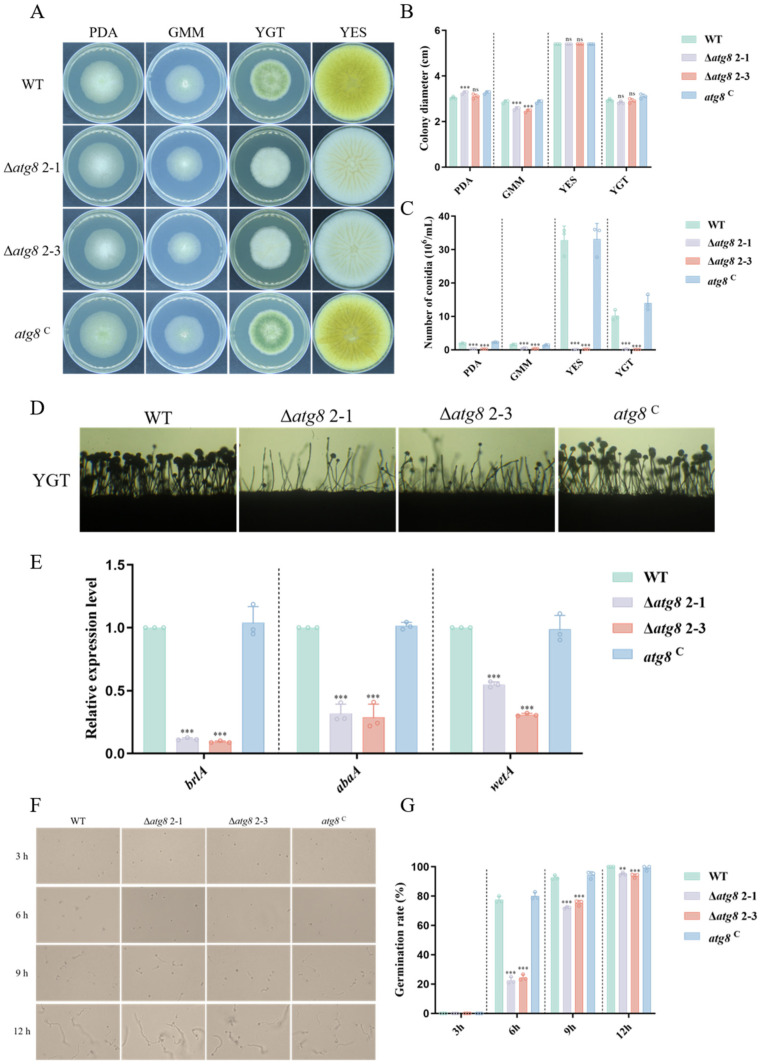
Effect of ATG8 on growth and conidia of *A. flavus*. (**A**) The growth of WT, Δ*atg8*, and *atg8*^C^ strains on PDA, GMM, YES and YGT at 37 °C for 4 days in the dark; (**B**) Colonial diameter of WT, Δ*atg8*, and *atg8*^C^ strains on the mentioned media; (**C**) Conidia count of WT, Δ*atg8*, and *atg8*^C^ strains on the mentioned media; (**D**) Conidiophore morphology of WT, Δ*atg8*, and *atg8*^C^ strains following 12 h of cultivation in darkness at 37 °C; (**E**) Relative expression levels of the conidium-related genes *brlA*, *abaA*, and *wetA* in WT, Δ*atg8*, and *atg8*^C^ strains; (**F**) Conidium germination of WT, Δ*atg8*, and *atg8*^C^ strains under dark conditions at 37 ◦C for 3, 6, 9, and 12 h; (**G**) Conidium germination rate of WT, Δ*atg8*, and *atg8*^C^ strains at 3, 6, 9, and 12 h. **, *** and ns indicate *p* < 0.01, *p* < 0.001 and not significant, respectively.

To investigate the impact of ATG8 on hyphal growth and conidiation in *A. flavus*, wild-type (WT) and mutant strains were cultured on various media (PDA, GMM, YES, and YGT) at 37 °C for 4 days. While colony growth of Δ*atg8* was notably slower on GMM compared to WT and *atg8*^C^, colony diameter remained unchanged for Δ*atg8* mutants when cultured on PDA, YES, and YGT (Figure 2A,B). Additionally, Figure 2C illustrated a significant decrease in spore production in the Δ*atg8* strain compared to WT and *atg8*^C^ across the four different culture media mentioned above. Microscopic examination revealed sparser conidiophores and smaller conidium heads in Δ*atg8* compared to WT, with conidiophore morphology of *atg8*^C^ resembling that of WT (Figure 2D). Further assessment of conidia germination rates demonstrated significantly lower rates for Δ*atg8* compared to WT and *atg8*^C^ at the 6th, 9th, and 12th hours (Figure 2F,G), indicating inhibition of spore germination due to *atg8* deletion in *A. flavus*. Additionally, the measurement of expression levels of conidium-related genes (*brlA*, *abaA* and *wetA*) showed a sharp decrease in Δ*atg8* mutants compared to WT and *atg8*^C^ (Figure 2E). Collectively, these findings confirm the significance of ATG8 in *A. flavus* development and sporulation formation.

### 3.4. ATG8 Contributes to Sclerotia Formation

**Figure 3:**
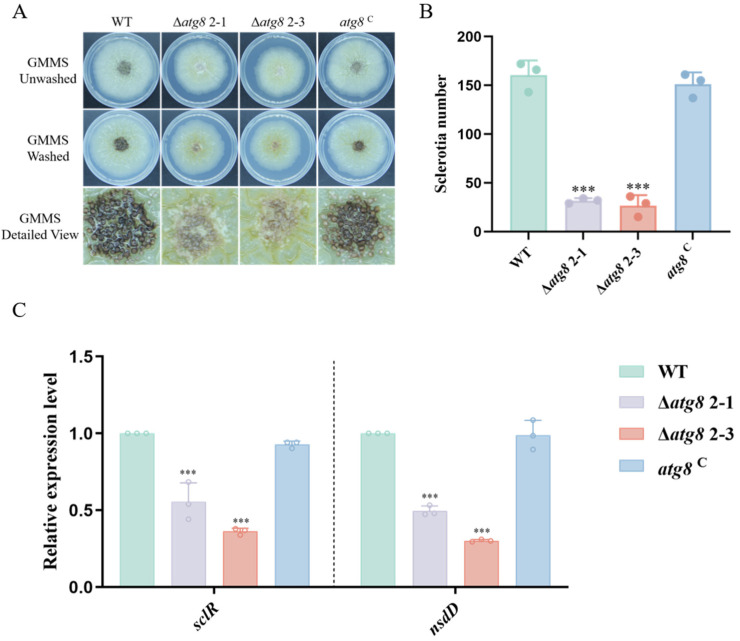
Sclerotium production of WT, Δ*atg*8, and *atg8*^C^ strains. (**A**) Sclerotium development of WT, Δ*atg*8, and *atg8*^C^ strains observed in GMMS medium at 37 °C for 5 days; (**B**) Quantification of sclerotia formation in WT, Δ*atg*8, and *atg8*^C^ strains cultured on GMMS medium; (**C**) Comparison of relative expression levels of sclerotium-related genes (*sclR* and *nsdD*) among WT, Δ*atg*8, and *atg8*^C^ strains. *** indicates *p* < 0.001.

*A. flavus* relies on the production of sclerotia to survive adverse environmental conditions [33]. To investigate the importance of ATG8 in the sclerotium formation, WT and mutant strains were inoculated on GMMS media at 37 °C for 5 days. Figure 3A,B revealed a significant reduction in the number of sclerotia in Δ*atg8* compared to WT and *atg8*^C^ strains. Further RT-qPCR demonstrated that the expression of sclerotium-related genes (*sclR* and *nsdD*) in the Δ*atg8* mutants significantly decreased in comparison with those in WT and *atg8*^C^ (Figure 3C). These findings suggest that ATG8 is indispensable for sclerotium development in *A. flavus*.

### 3.5. ATG8 Regulates Aflatoxin Biosynthesis in A. flavus

**Figure 4:**
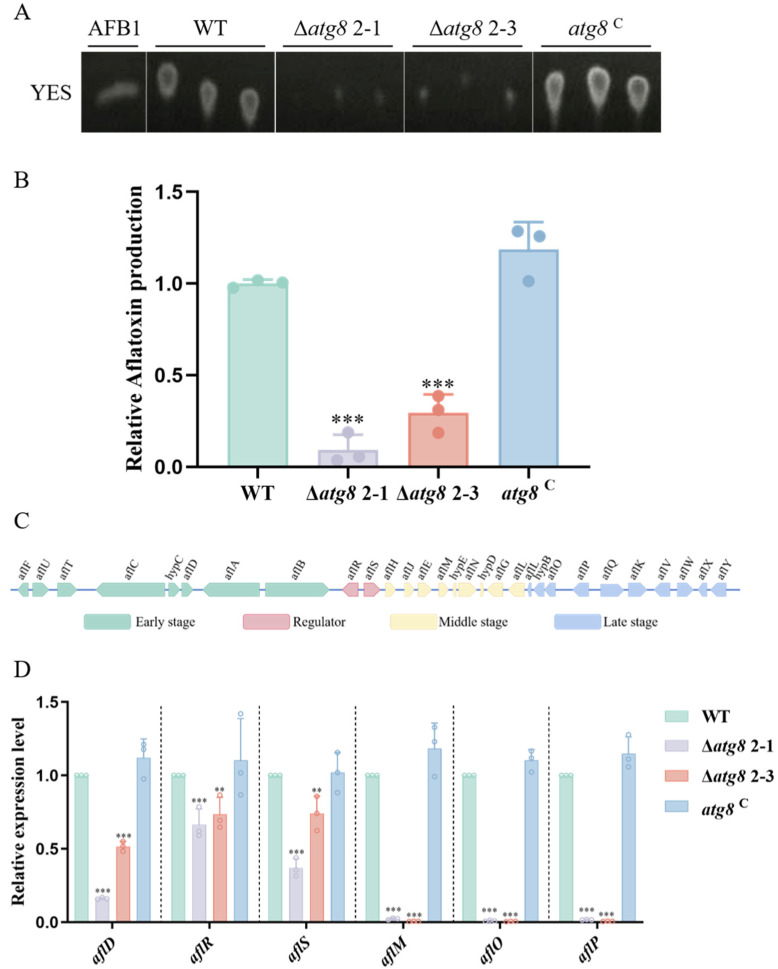
ATG8 contributes to aflatoxin biosynthesis. (**A**) Thin-layer chromatography analysis of AFB1 production by WT, Δ*atg*8, and *atg8*^C^ strains in YES liquid media after 5 days of culture at 29 °C; (**B**) Quantification analysis of AFB1 production in panel A; (**C**) AF gene cluster in *A. flavus* categorized into three stages; (**D**) Relative expression levels of genes related to AF biosynthesis. ** and *** indicate *p* < 0.01 and *p* < 0.001, respectively.

Aflatoxin B1 (AFB1), a notorious mycotoxin produced by *A. flavus*, contaminates different crops such as cotton and maize, and causes immense effects on the health of humans and animals [34,35]. In order to reveal the effect of ATG8 on the AFB1 biosynthesis, we measured AFB1 production in WT, Δ*atg8*, and *atg8*^C^ strains. TLC assays revealed that the Δ*atg8* mutant accumulated less AFB1 than the WT and *atg8*^C^ strains when cultured in YES media (Figure 4A,B). There are 29 genes associated with AF biosynthesis, stages: early, middle, and late stages (Figure 4C). Moreover, we evaluated the expression levels of AF-related genes, which were significantly downregulated as depicted in Figure 4D. The results demonstrated that Δ*atg8* mutant strain disrupted the ability of *A. flavus* to produce AFB1.

### 3.6. Response of ATG8 to Multiple Stresses in A. flavus

**Figure 5:**
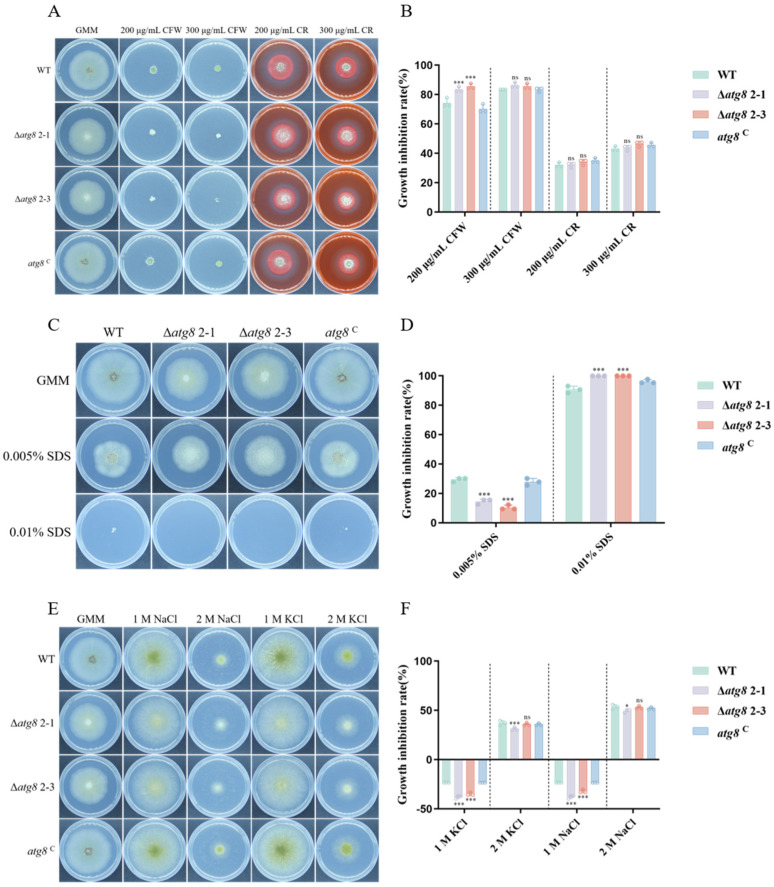
ATG8 involvement in *A. flavus* response to environmental stresses. (**A**) Fungal strains exposed to cell wall stress induced by CFW and CR on PDA for 4 days; (**B**) Growth inhibition rates under cell wall stress caused by CFW and CR; (**C**) Fungal strains under cell membrane stress induced by SDS on PDA for 4 days; (**D**) Growth inhibition rates under cell membrane stress caused by SDS; (**E**) Fungal strains under osmotic stress induced by KCl and NaCl on PDA for 4 days; (**F**) Growth inhibition rates under osmotic stress caused by KCl and NaCl. *, *** and ns indicate *p* < 0.05, *p* < 0.001 and not significant, respectively.

To characterize the role of ATG8 in fungal susceptibility to environmental stresses, we treated the Δ*atg8* with various inhibitors. Figure 5A,B show that the Δ*atg8* mutant exhibited heightened sensitivity to 200 μg/mL CFW, a cell wall stress agent. Furthermore, compared to the WT and *atg8*^C^ strains, the sensitivity of Δ*atg8* mutant to 300 μg/mL CFW, 200 and 300 μg/mL CR, also known as a cell wall stress agent, increased. Then, we determined the sensitivity of Δ*atg8* mutant to a cell membrane stress agent. Adding SDS to PDA media revealed a downregulation in sensitivity to 0.005% SDS for the Δ*atg8* mutant. In contrast, the Δ*atg8* mutant exhibited heightened sensitivity to 0.01% SDS compared to the WT and *atg8*^C^ strains (Figure 5C,D). Furthermore, we examined the response of *A. flavus* to hyperosmotic stress following ATG8 deletion by supplementing PDA media with KCl or NaCl. As shown in Figure 5E,F, the sensitivity of the Δ*atg8* mutant decreased at all tested concentrations of KCl or NaCl. The abovementioned data indicate the involvement of ATG8 in responding to these stress stimuli in *A. flavus*.

**Figure 6:**
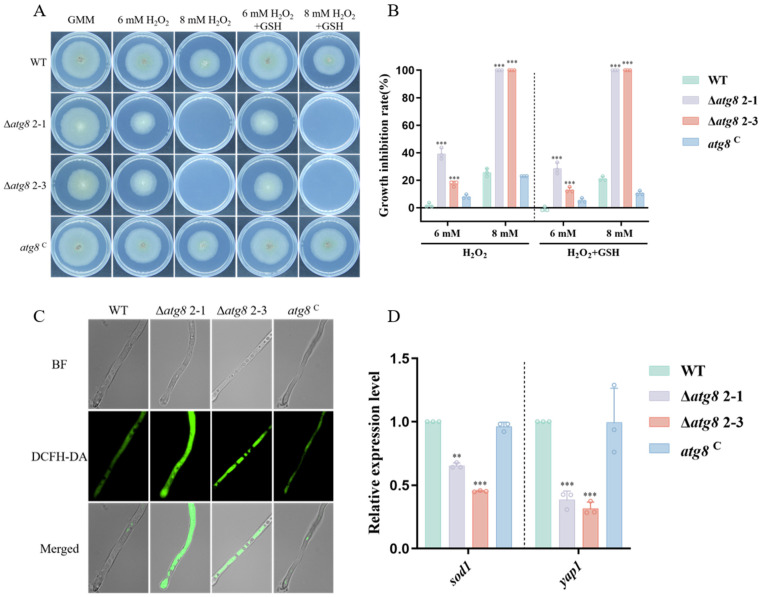
ATG8’s role in resistance to reactive oxygen species. (**A**) *atg8* deletion increases sensitivity to hydrogen peroxide (H_2_O_2_); (**B**) Growth inhibition rates under H_2_O_2_; (**C**) Fluorescence microscopy analysis of intracellular ROS in the mycelia of *atg8* mutants. Intracellular ROS was visualized by DCFH-DA; (**D**) Relative expression levels of *sod1* and *yap1* in WT, Δ*atg8*, and *atg8*^C^ strains. ** and *** indicate *p* < 0.01 and *p* < 0.001, respectively.

In fungi, autophagy has been recognized as essential for oxidative stress resistance [36]. Therefore, the resistance ability of Δ*atg8* mutant against oxidative stress was measured. Sensitivity assays on PDA revealed that Δ*atg8* was very sensitive to the oxidative agent H_2_O_2_ (Figure 6A,B). To further confirm that autophagy is involved in resistance to reactive oxygen species (ROS) in *A. flavus*, we cultured the strains in PDB at 37 °C for 12 h and observed them under fluorescence microscopy. The Δ*atg8* hyphae, stained with DCFH-DA, emitted significantly brighter green fluorescence compared to WT and *atg8*^C^ strains, indicating a higher accumulation of ROS in the Δ*atg8* strain (Figure 6C). RT-qPCR analyses also revealed significantly decreased expression levels of *sod1* and *yap1* in Δ*atg8* (Figure 6D). These data indicate the involvement of autophagy in antioxidant stress systems.

### 3.7. Effect of ATG8 Mutants on Pathogenicity to Crop Seeds

**Figure 7:**
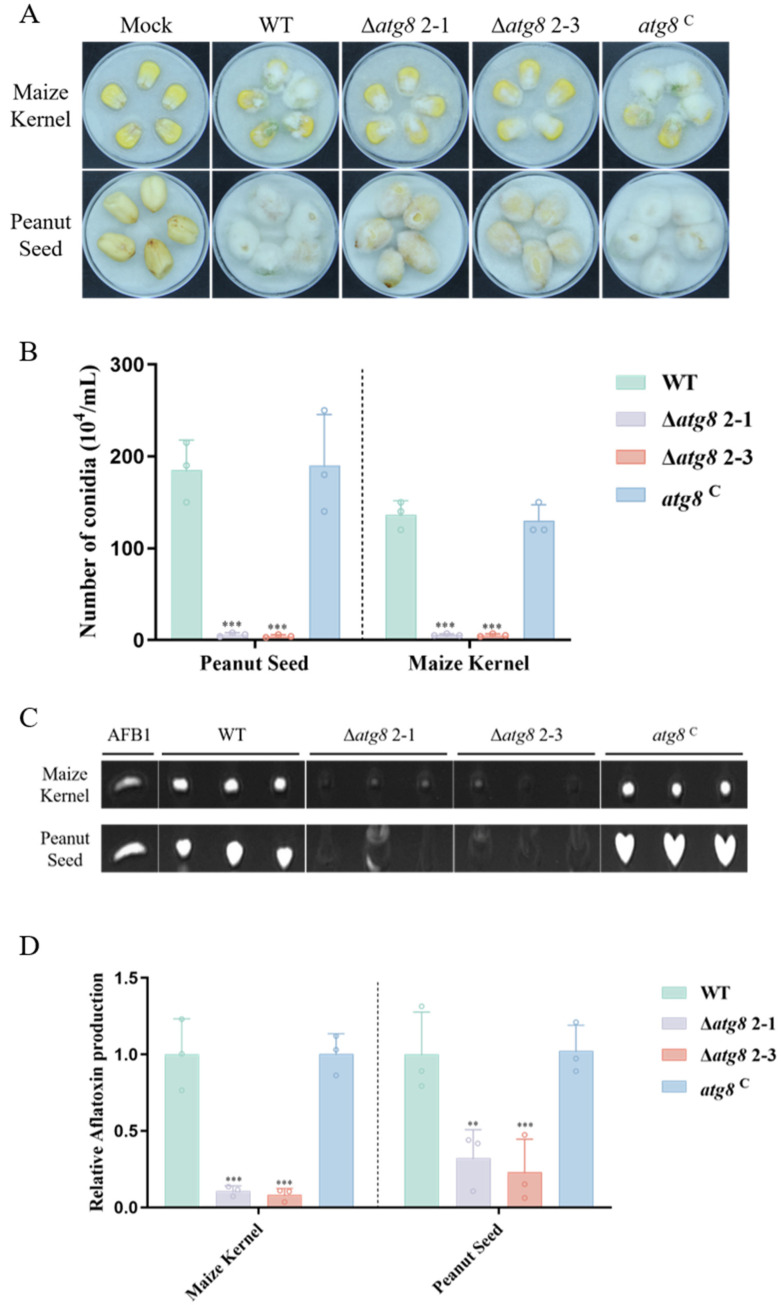
ATG8’s role in *A. flavus* colonization on crops. (**A**) Colonization of peanut seeds and maize kernels by WT, Δ*atg8*, and *atg8*^C^ strains at 29 °C in darkness for 5 days; (**B**) Conidia count statistics of the fungal strains on peanut seeds and maize kernels; (**C**) TLC analysis of AFB1 yield by the fungal strains on the kernels; (**D**) Quantification analysis of AFB1 production in panel C. ** and *** indicate *p* < 0.01 and *p* < 0.001, respectively.

*A. flavus* is known to produce aflatoxins on various crops, including corn, peanuts, cottonseed, and nuts, resulting in significant agricultural economic losses and posing health risks [37,38]. The pathogenicity of ATG8 on crop seeds was detected. The result indicated that the Δ*atg8* mutant exhibited reduced mycelia vigor compared to the WT and *atg8*^C^ strains (Figure 7A). We further detected that in the conidia production from the infected crop seed, as shown in Figure 7B, compared to the WT and *atg8*^C^ strain, the conidia production of Δ*atg8* mutant is significantly reduced. All the above results suggested that the Δ*atg8* mutant is impaired in both infection and sporulation on crop seeds. Additionally, AFB1 production in infected crop seeds was quantified using TLC, and the results showed that the Δ*atg8* mutant produced little detectable AFB1 in the maize kernels and peanut seed compared to the WT and *atg8*^C^ strains (Figure 7C,D). These results underscore the crucial role of ATG8 in crop infection by *A. flavus*.

## 4. Discussion

Autophagy is a highly conserved evolutionary process where proteins, membranes, and organelles are broken down and repurposed to sustain energy balance within eukaryotic cells [39,40]. In this study, the ATG8, a marker to monitor autophagosome formation [36], was characterized in *A. flavus*. We identified the *atg8* gene and found that the ATG8 protein plays a crucial role in various aspects of *A. flavus* biology, including vegetative growth, conidial development, pathogenicity, resistance to ROS, and AFB1 biosynthesis.

Autophagy is crucial for fungi, influencing their growth, morphology, and development. Studies in various fungi, such as *S. cerevisiae* [41], *F. verticillioides* [42], and *A. alternata* [36], have shown that mutations or deletions in the ATG8 gene lead to developmental defects or reduced vegetative growth. Our studies were consistent with recent research on ATG8 functions in fungi. Specifically, our study reveals that Δ*atg8* mutants exhibit similar defects, with the deletion of *atg8* resulting in reduced vegetative growth and developmental abnormalities in conidia. These findings collectively support the idea of the conserved roles of ATG8 in regulating cellular differentiation in both yeast and filamentous fungi.

Previous research has highlighted the crucial involvement of autophagy in pathogenicity [43,44]. Histone acetyltransferase acetylates autophagy-related proteins, regulating both appressorium formation and pathogenicity in *M. oryzae* [39]. Furthermore, inhibiting autophagy may decrease the pathogenicity of *F. graminearum* [30,45]. In *Colletotrichum fructicola*, deletion of the *cfatg8* and *cfatg9* genes impaired appressorium function and caused defects in pathogenicity [46]. Our study further demonstrates that Δ*atg8* mutants in *A. flavus* exhibit pathogenicity defects. The reduced pathogenicity of Δ*atg8* on maize kernels and peanut seeds may arise from various phenotypic abnormalities, including reduced hyphal growth and developmental defects of conidia. These findings highlight the significance of autophagy in pathogenicity.

The ability of fungi to infect hosts is closely linked to their capacity to withstand diverse environmental stresses. To investigate the involvement of ATG8 in *A. flavus* response to various environmental stresses, inhibitors such as KCl, NaCl, SDS, CFW, and CR were tested. The results indicate that ATG8 plays a significant role in *A. flavus* response to environmental stresses, including osmotic stress, cell membrane stress, and cell wall stress (Figure 5). The fluorescence microscopy showed that deletion of *atg8* leads to the accumulation of high ROS levels. Furthermore, we observed downregulation of genes involved in ROS detoxification in the Δ*atg8* mutant (Figure 6). Consistent with our results, in *A. alternata*, the Δ*AaAtg8* failed to detoxify ROS effectively, resulting in ROS accumulation [25]. Overall, our findings suggest that autophagy-mediated ROS detoxification plays a critical role in the oxidative stress response.

Aflatoxins have acutely toxic, immunosuppressive, carcinogenic, and teratogenic effects [47,48,49]; therefore, the prevention and control of aflatoxin are crucial. Our study revealed a striking decrease in AFB1 production in the Δ*atg8* mutant compared to the WT and *atg8*^C^ strains, indicating that autophagy is necessary for AFB1 biosynthesis in *A. flavus*. Furthermore, RT-qPCR analysis revealed a downregulation of aflatoxin biosynthesis gene expression in the Δ*atg8* mutant. It is speculated that ATG8 regulates AFB1 biosynthesis by controlling the gene cluster responsible for aflatoxin biosynthesis in *A. flavus*.

In summary, we identified the ATG8 protein and constructed *atg8* deletion and complementation mutants using a homologous recombination strategy. We proposed that ATG8 is vital for the growth, conidial development, stress resistance, AFB1 biosynthesis, and pathogenicity of *A. flavus*. Given the crucial role of ATG8 in maintaining autophagy and pathogenicity, future investigations will explore ATG8-interacting proteins to enhance our comprehension of the autophagy regulatory network in *A. flavus*.

## Figures and Tables

**Table 1:** *Aspergillus* strains used in this study.

Name of Strain	Genotype	Background	Source
NRRL 3357	*A. flavus* Wild-type	NRRL 3357	[31]
TJES20.1	*pyrG1*, Δ*ku70*, Δ*argB*::*AfpyrG*	NRRL 3357	[31]
TXZ 21.3	*pyrG1*, Δ*ku70*, Δ*argB*	NRRL 3357	[31]
Δ*atg8*	*pyrG1*, Δ*ku70*, Δ*argB*::*AfpyrG*, Δ*atg8*::*argB*	NRRL 3357	This study
Δ*atg8*Δ*pyrG*	*pyrG1*, Δ*ku70*, Δ*atg8*::*argB*	NRRL 3357	This study
*atg8* ^C^	*pyrG1*, Δ*ku70*, Δ*atg8*::*argB*, *atg8*::*AfpyrG*	NRRL 3357	This study

**Table 2:** Gene-specific primers used for deleted and complemented mutants.

Primers	Sequence (5′-3′)	Application
*atg8*/P1	TGAGAAGTGGCAGAGTGAC	*atg8* deletion
*atg8*/P2	TGTCCCAGTTCCTGCTTAGT	
*atg8*/P3	TTCTACCGAACTCATCACCACCGGGAAACAGTAGGAAGAAGGTGGAT	
*atg8*/P4	TGGTGCCCGCATTCACATGTCACGGGCTTCGCCGTCAGTATGTGTT	
*atg8*/P5	AGTCAGTCATCGCCTGTTTT	
*atg8*/P6	GCGGTTCCTGGTGGGTTAT	
*argB*/F	TCCCGGTGGTGATGAGTTC	*A. flavus argB*
*argB*/R	CCCGTGACATGTGAATGCG	
*pyrG*/F	GCCTCAAACAATGCTCTTCACCC	*A. fumigatus pyrG*
*pyrG*/R	GTCTGAGAGGAGGCACTGATGC	
*atg8*-orf/F	CAACTCTATCTGATCCGTAC	*atg8* mutant screen
*atg8*-orf/R	GAGACTATGTCAATATGTGCC	
*argB*-170/R	TGTCCAGTTCGGGTTAGCG	
*argB*-1205/F	ACGGTGTCTCAAAGCCAGG	
*pyrG*-801/R	CAGGAGTTCTCGGGTTGTCG	
*pyrG*-351/F	CAGAGTATGCGGCAAGTCA	
c-*atg8*/p1	GTCTGCGCTGAGAAGTGG	*atg8* complementation
c-*atg8*/P2	GAAATAGAGGTAGCCTAATCG	
c-*atg8*/P3	GGGTGAAGAGCATTGTTTGAGGCTTAAAGATCGCCGAAGGTG	
c-*atg8*/P4	GCATCATGCCTCCTCTCAGACTCCCGGTGGTGATGAGTTC	
c-*atg8*/P5	GACCCAAACTGTCAGAGC	
c-*atg8*/P6	ACCCAGGCAATCTTGAGGC	

**Table 3:** Gene-specific primers used for RT-qPCR.

Primers	Sequence (5′-3′)	Application
*atg8*/QF	GACATCGCCACTATTGATAAG	*atg8* RT-qPCR
*atg8*/QR	GTTCTTCGTAGATGCTGCTC	
*abaA*/QF	ACGGAAATCGCCAAAGACA	*abaA* RT-qPCR
*abaA*/QR	CCGGAATTGCCAAAGTAGG	
*brlA*/QF	CTCCAGCGTCAACCTTCA	*brlA* RT-qPCR
*brlA*/QR	TCAAATGCTCTTGCCTCTTA	
*wetA*/QF	GGCGTCTAGTTGTCAGGAG	*wetA* RT-qPCR
*wetA*/QR	ACATTCATTGAGTTGGAGGA	
*sclR*/QF	CAATGAGCCTATGGGAGTGG	*sclR* RT-qPCR
*sclR*/QR	ATCTTCGCCCGAGTGGTT	
*nsdD*/QF	GGACTTGCGGGTCGTGCTA	*nsdD* RT-qPCR
*nsdD*/QR	AGAACGCTGGGTCTGGTGC	
*aflD*/QF	GCTCCCGTCCTACTGTTTC	*aflD* RT-qPCR
*aflD*/QR	CATGTTGGTGATGGTGCTG	
*aflR*/QF	AAAGCACCCTGTCTTCCCTAAC	*aflR* RT-qPCR
*aflR*/QR	GAAGAGGTGGGTCAGTGTTTGTAG	
*aflS*/QF	CGAGTCGCTCAGGCGCTCAA	*aflS* RT-qPCR
*aflS*/QR	GCTCAGACTGACCGCCGCTC	
*aflM*/QF	CCCCAGAAGAATTTGACCG	*aflM* RT-qPCR
*aflM*/QR	ACGCAAGCAGTGTTAGAGC	
*aflO*/QF	GATTGGGATGTGGTCATGCGATT	*aflO* RT-qPCR
*aflO*/QR	GCCTGGGTCCGAAGAATGC	
*aflP*/QF	ACGAAGCCACTGGTAGAGGAGATG	*aflP* RT-qPCR
*aflP*/QR	GTGAATGACGGCAGGCAGGT	
*sod1*/QF	ATGGTCAAGGCTGGTAGG	*sod1* RT-qPCR
*sod1*/QR	CAGTGATAGGCTGGGAGG	
*yap1*/QF	CTTCTTCTTGCCGCTCTT	*yap1* RT-qPCR
*yap1*/QR	TCCGTAACCCAATCCACC	
*actin*/QF	ACGGTGTCGTCACAAACTGG	*actin* RT-qPCR
*actin*/QR	CGGTTGGACTTAGGGTTGATAG	

## Data Availability

Data are contained within the article.
